# Supplementary material for: Rotational symmetry breaking in superconducting nickelate Nd0.8Sr0.2NiO2 films
Source: Nat Commun. 2023 Nov 7;14:7155. doi: 10.1038/s41467-023-42988-8 (PMC10630465; doi:10.1038/s41467-023-42988-8)
Supplement: Supplementary file 1 — Supplementary Information [file 41467_2023_42988_MOESM1_ESM.pdf]

Supplementary Information for

"Rotational symmetry breaking in superconducting nickelate $Nd_{0.8}Sr_{0.2}NiO_2$ films"

Haoran Ji[1#], Yi Liu[2,3#], Yanan Li[1,4#], Xiang Ding[5], Zheyuan Xie[1], Chengcheng Ji[1], Shichao Qi[1], Xiaoyue Gao[1,6], Minghui Xu[5], Peng Gao[1,6], Liang Qiao[5*], Yi-feng Yang[7,8,9], Guang-Ming Zhang[10,11] and Jian Wang[1,12,13,14,15*]

[1]International Center for Quantum Materials, School of Physics, Peking University, Beijing 100871, China

[2]Department of Physics and Beijing Key Laboratory of Opto-electronic Functional Materials & Micro-nano Devices, Renmin University of China, Beijing 100872, China

[3]Key Laboratory of Quantum State Construction and Manipulation (Ministry of Education), Renmin University of China, Beijing 100872, China

[4]Department of Physics, The Pennsylvania State University, University Park, Pennsylvania 16802, USA

[5]School of Physics, University of Electronic Science and Technology of China, Chengdu 610054, China

[6]Electron Microscopy Laboratory, School of Physics, Peking University, Beijing 100871, China

[7]Beijing National Laboratory for Condensed Matter Physics and Institute of Physics, Chinese Academy of Sciences, Beijing 100190, China

[8]School of Physical Sciences, University of Chinese Academy of Sciences, Beijing 100049, China

[9]Songshan Lake Materials Laboratory, Dongguan, Guangdong 523808, China

[10]State Key Laboratory of Low-Dimensional Quantum Physics and Department of Physics, Tsinghua University, Beijing 100084, China

[11]Frontier Science Center for Quantum Information, Beijing 100084, China

[12]Collaborative Innovation Center of Quantum Matter, Beijing 100871, China

[13]CAS Center for Excellence in Topological Quantum Computation, University of Chinese Academy of Sciences, Beijing 100190, China
[14]Beijing Academy of Quantum Information Sciences, Beijing 100193, China
[15]Hefei National Laboratory, Hefei 230088, China

[#]These authors contribute equally: Haoran Ji, Yi Liu, Yanan Li.
[*]Corresponding author. jianwangphysics@pku.edu.cn (J.W.), liang.qiao@uestc.edu.cn (L.Q.).

I. Sample characterizations

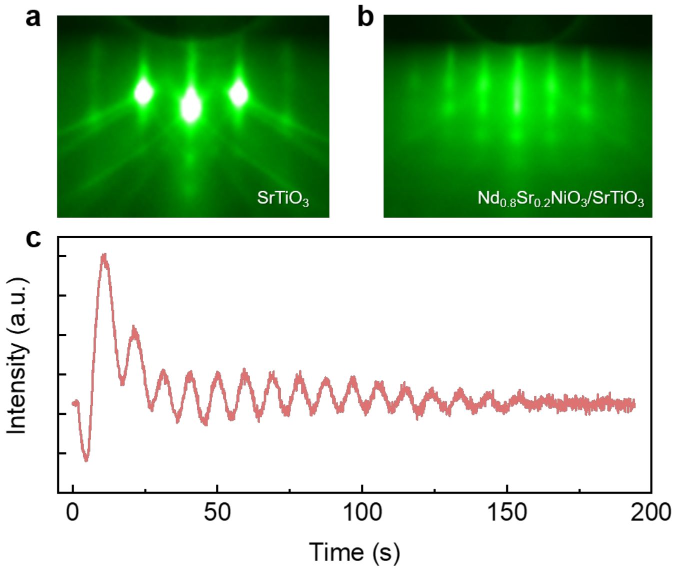

Fig. S1. Reflection high-energy electron diffraction (RHEED) results of the $Nd_{0.8}Sr_{0.2}NiO_3$ sample. **a**, **b**, RHEED patterns of $SrTiO_3$ (001) substrate before growth of $Nd_{0.8}Sr_{0.2}NiO_3$ film (**a**) and $Nd_{0.8}Sr_{0.2}NiO_3/SrTiO_3$ (001) after the film growth (**b**). **c**, Oscillation curves of the RHEED intensity monitored during the growth of $Nd_{0.8}Sr_{0.2}NiO_3$ on $SrTiO_3$ (001). The RHEED patterns demonstrate that the $Nd_{0.8}Sr_{0.2}NiO_3$ films were grown in a layer-by-layer mode.

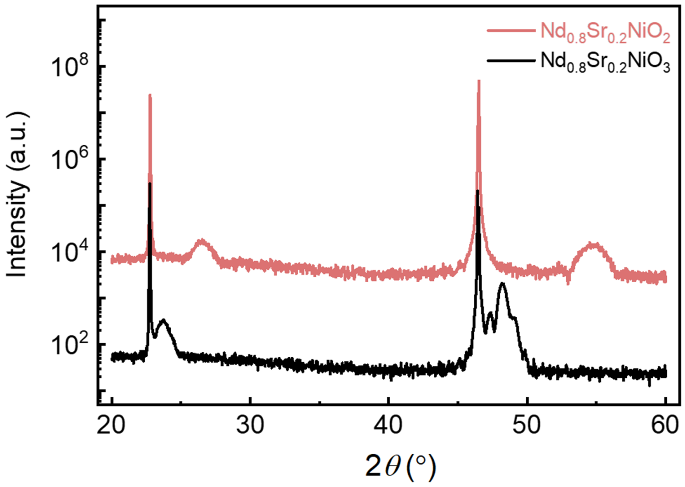

Fig. S2. X-ray diffraction (XRD) data for the $Nd_{0.8}Sr_{0.2}NiO_3$ and the superconducting $Nd_{0.8}Sr_{0.2}NiO_2$ thin films. XRD patterns of the 15-nm-thick $Nd_{0.8}Sr_{0.2}NiO_3$ (001) film grown on $SrTiO_3$ (001) substrate and the $Nd_{0.8}Sr_{0.2}NiO_2$ film after reduction.

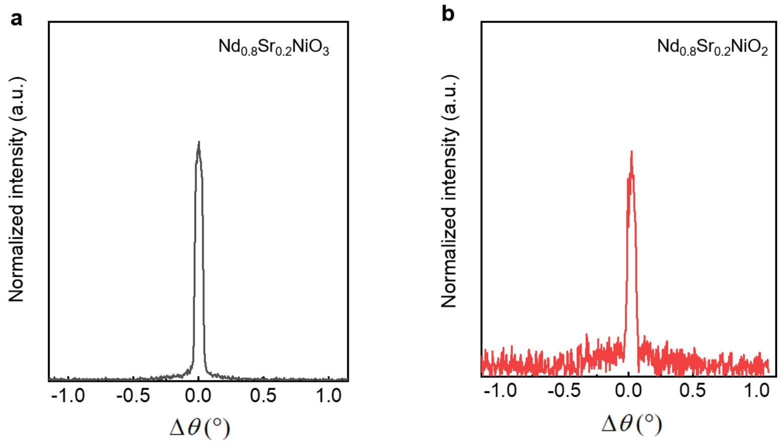

Fig. S3. Rocking curve scans across the (002) diffraction peak for the $Nd_{0.8}Sr_{0.2}NiO_3$ and the superconducting $Nd_{0.8}Sr_{0.2}NiO_2$ thin films. Rocking curve scans of the 15-nm-thick $Nd_{0.8}Sr_{0.2}NiO_3$ (001) film (**a**) and the $Nd_{0.8}Sr_{0.2}NiO_2$ film (**b**). The full width at half maximum of the rocking curves is around 0.1°, demonstrating the high crystalline quality of the films.

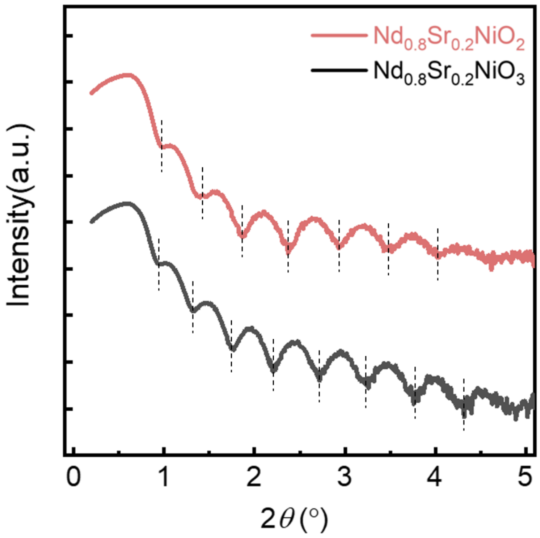

Fig. S4. X-ray reflectivity (XRR) data for the $Nd_{0.8}Sr_{0.2}NiO_3$ and the superconducting $Nd_{0.8}Sr_{0.2}NiO_2$ thin films. XRR patterns of the 15-nm-thick $Nd_{0.8}Sr_{0.2}NiO_3$ (001) film grown on $SrTiO_3$ (001) substrate and the $Nd_{0.8}Sr_{0.2}NiO_2$ film after reduction. The change of the oscillation period from $Nd_{0.8}Sr_{0.2}NiO_3$ (~ 0.49°) to $Nd_{0.8}Sr_{0.2}NiO_2$ (~ 0.51°) represents the change of the crystal structure after the topotactic reduction procedure.

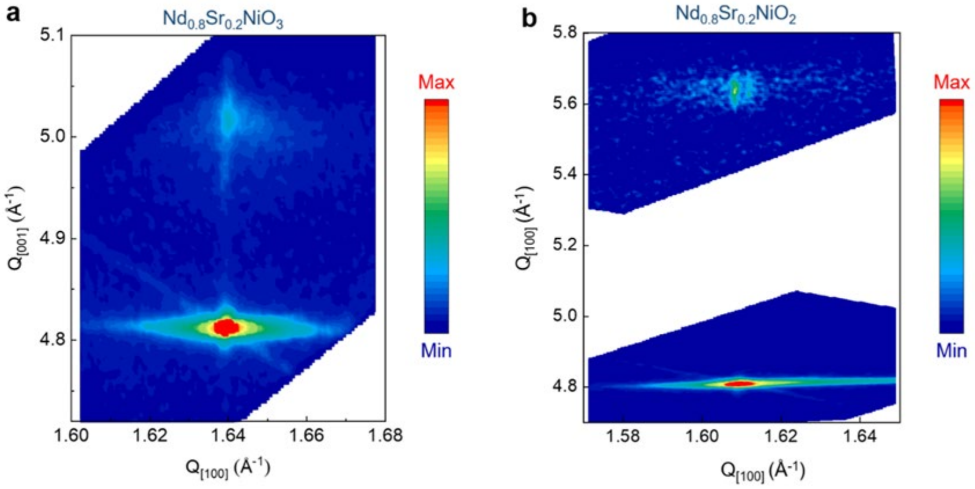

Fig. S5. Reciprocal space mapping (RSM) for the $Nd_{0.8}Sr_{0.2}NiO_3$ and the superconducting $Nd_{0.8}Sr_{0.2}NiO_2$ thin films. a, **b**, RSM of the diffraction peak from the 15-nm-thick $Nd_{0.8}Sr_{0.2}NiO_3$ (001) film grown on $SrTiO_3$ (001) substrate (**a**) and the $Nd_{0.8}Sr_{0.2}NiO_2$ film after reduction (**b**). The RSM shows that the film and the substrate exhibit the same $Q_{[100]}$, indicating the $Nd_{0.8}Sr_{0.2}NiO_3$ and the $Nd_{0.8}Sr_{0.2}NiO_2$ films are

fully strained with the $SrTiO_3$ substrate.

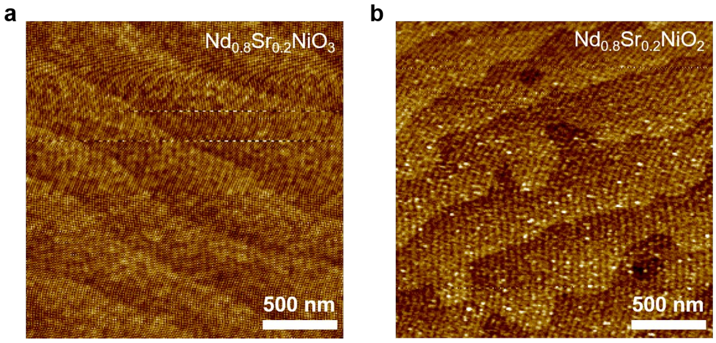

Fig. S6. Atomic force microscope images for the $Nd_{0.8}Sr_{0.2}NiO_3$ and the superconducting $Nd_{0.8}Sr_{0.2}NiO_2$ thin films. Atomic Force Microscope images of the 15-nm-thick $Nd_{0.8}Sr_{0.2}NiO_3$ (001) film grown on $SrTiO_3$ (001) substrate and the $Nd_{0.8}Sr_{0.2}NiO_2$ film after reduction, demonstrating the high quality of the samples.

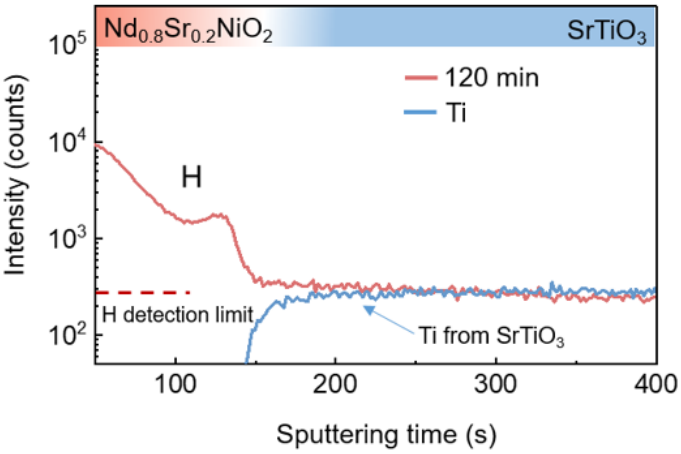

Fig. S7. Secondary-ion mass spectrometry (SIMS) of the $Nd_{0.8}Sr_{0.2}NiO_2$ film on the $SrTiO_3$ substrate. SIMS depth-profile of H element of the $Nd_{0.8}Sr_{0.2}NiO_2$ film grown on $SrTiO_3$ (001) substrate. The Ti signals from the $SrTiO_3$ (001) substrate is used to mark the interface position (blue curves). The H detection limit is determined by the as-received $SrTiO_3$ (001) substrate (red dashed line). The intensity of the H element in the $Nd_{0.8}Sr_{0.2}NiO_2$ film is orders of magnitude higher than the background, demonstrating the existence of abundant H in the film.

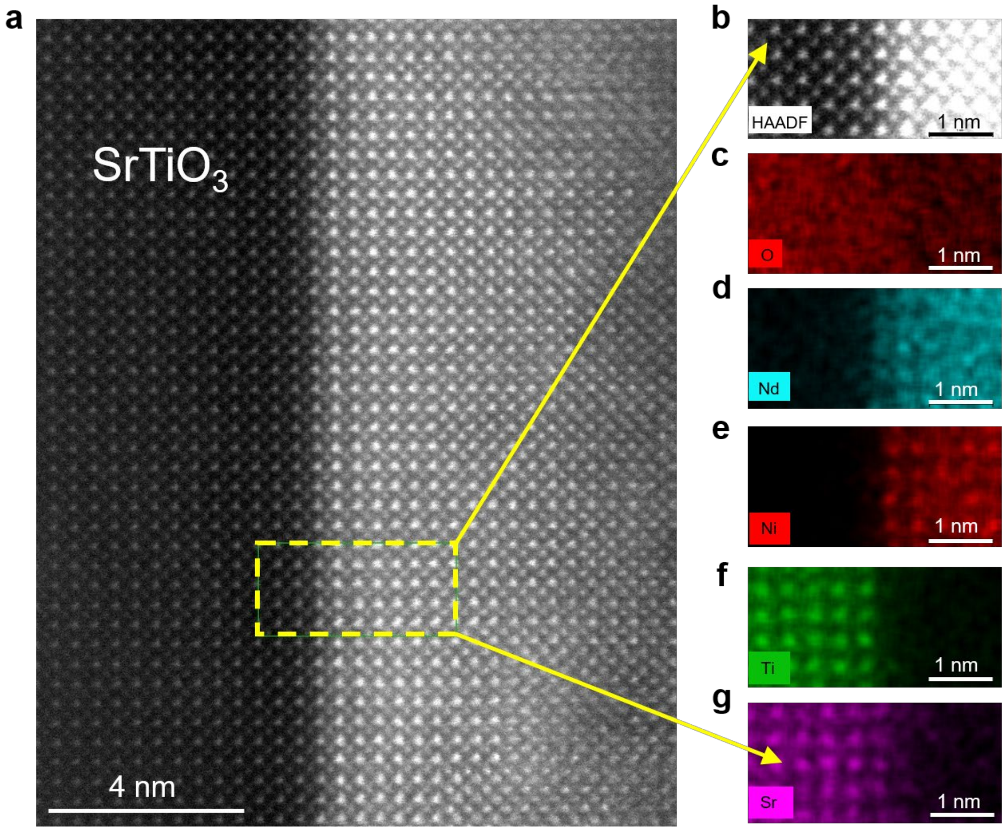

Fig. S8. Atomically resolved high-angle annular dark-field scanning transmission electron microscopy (HAADF-STEM) images and the elemental mapping of the $Nd_{0.8}Sr_{0.2}NiO_2/SrTiO_3$ interface. a-b, Atomically resolved STEM images of the $Nd_{0.8}Sr_{0.2}NiO_2/SrTiO_3$ interface, **c-g**, Energy dispersive spectrometer (EDS) maps for O (**c**), Nd (**d**), Ni (**e**), Ti (**f**), Sr (**g**) elements. The right panel is obtained from the yellow boxed area labelled in **a**. The atomically resolved STEM images show clearly that the $Nd_{0.8}Sr_{0.2}NiO_2$ film is coherently connected with the $SrTiO_3$ substrate at the interface.

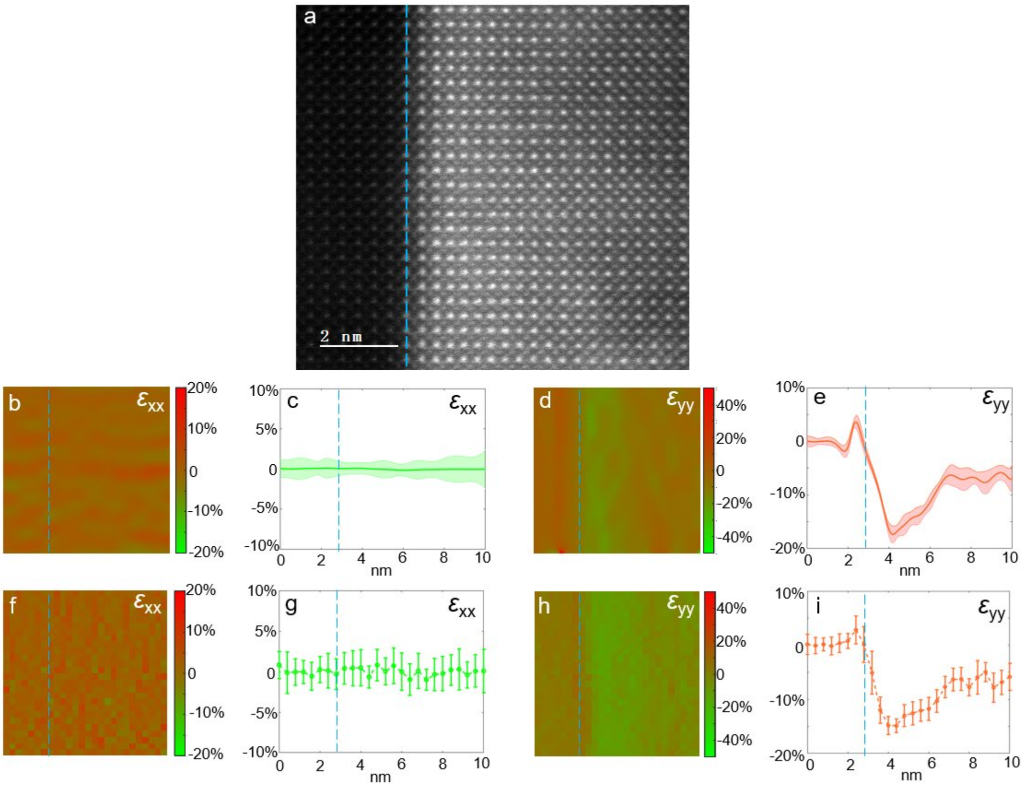

Fig. S9. Strain distribution of the $Nd_{0.8}Sr_{0.2}NiO_2/SrTiO_3$ interface area. a, HAADF–STEM image for $Nd_{0.8}Sr_{0.2}NiO_2/SrTiO_3$ interface. The low-contrast area corresponds to the $SrTiO_3$ substrate, and the high-contrast area corresponds to the $Nd_{0.8}Sr_{0.2}NiO_2$ thin film. **b**, **f**, In-plane strain ε_{xx} maps. **d**, **h**, Out-of-plane strain ε_{yy} maps. **b** and **d** are obtained via geometric phase analysis (GPA) of **a**. **f** and **h** are unit cell strain mappings calculated through fitting the atom position of **a**. **c**, **e**, **g**, **i**, Line profiles obtained by averaging the vertical intensity of **b, d**, **f, h**, respectively. Shade color regions in **c**, **e** and error bars in **g**, **i** are the standard deviations of pixels (**c** and **e**) or data (**g** and **i**) from the strain maps. The GPA and the atom position fitting results show the consistent strain distributions. The blue dashed lines mark the interface position.

II. Additional information for sample S1

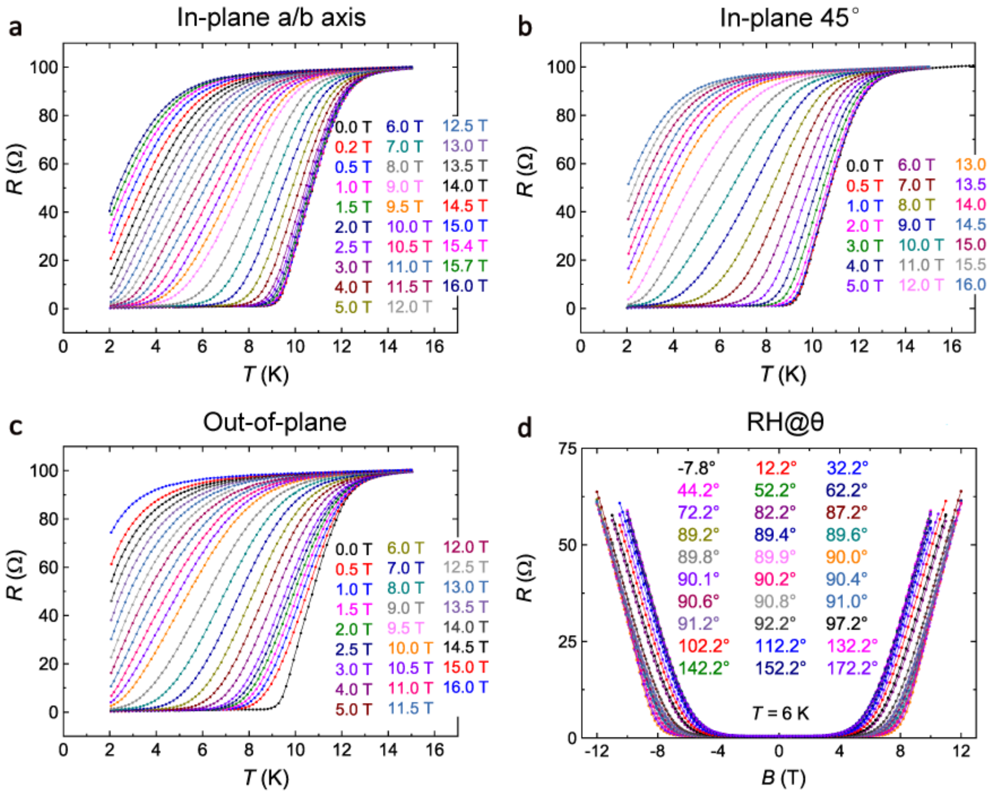

Fig. S10. *R*(*T*) and *R*(*H*) curves for the determination of $\boldsymbol{B_c}$. *R*(*T*) under different magnetic field applieds along the *a*/*b*-axis **(a)**, the *ab* diagonal direction **(b)**, and the *c*-axis **(c)**, **d**, *R*(*B*) at different θ angles at 6 K. The $B_{c//,0^o}(T)$, $B_{c//,45^o}(T)$, $B_{c\perp}(T)$ and $B_c(\theta)$ are determined using the 50% R_N criterion through the data shown in **a**, **b**, **c** and **d**, respectively.

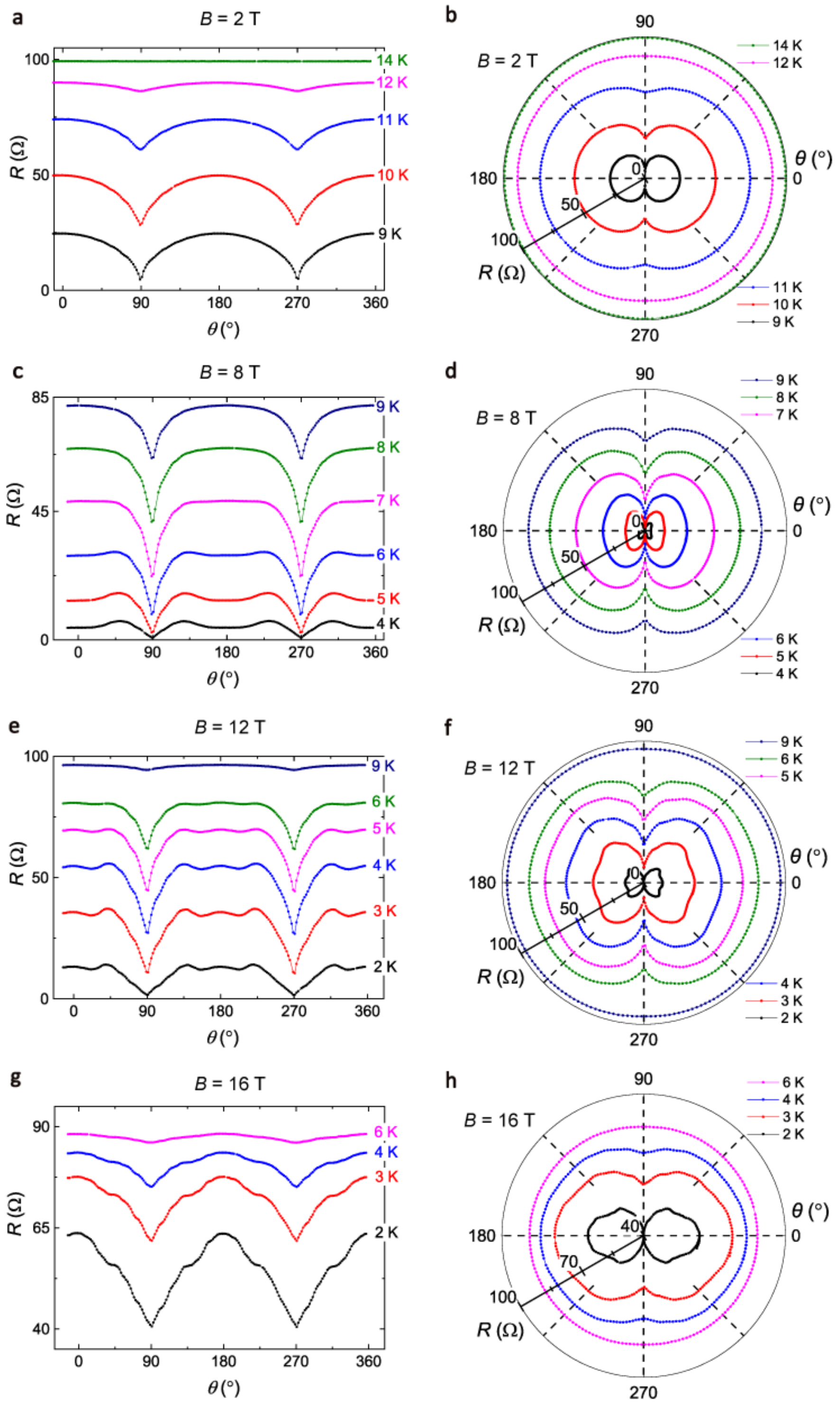

Fig. S11. *R*(*θ*) curves showing the quasi-2D anisotropy. Polar angular dependence of magnetoresistance $R(\theta)$ at different temperatures under 2 T (**a** and **b**), 8 T (**c** and **d**), 12 T (**e** and **f**) and 16 T (**g** and **h**). Here, θ represents the angle between the magnetic field and the *c*-axis of the $Nd_{0.8}Sr_{0.2}NiO_2$. The left panels show the rectangular plots and the right panels show the corresponding polar plots.

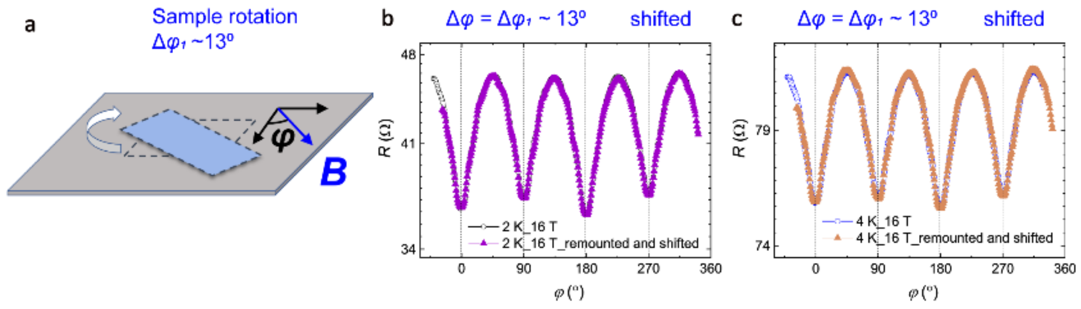

Fig. S12. Remounted measurements after an in-plane rotation Δφ_2. a, Schematic of the sample remounting after an in-plane rotation. **b**, the comparison between the initial results $R(\varphi)$ and the remeasured results $R(\varphi+\Delta\varphi_1)$ after an in-plane rotation $\Delta\varphi_1 \sim 13^o$ under 16 T at 2 K. **c**, The similar comparison between $R(\varphi)$ and $R(\varphi+\Delta\varphi_1)$ curves under 16 T at 4 K. In **b** and **c**, the $R(\varphi+\Delta\varphi_1)$ curves are shifted 13° and 1.6 Ω for comparison, which can nicely overlap with initial $R(\varphi)$ curves, confirming that the C_4+C_2 anisotropy is intrinsic.

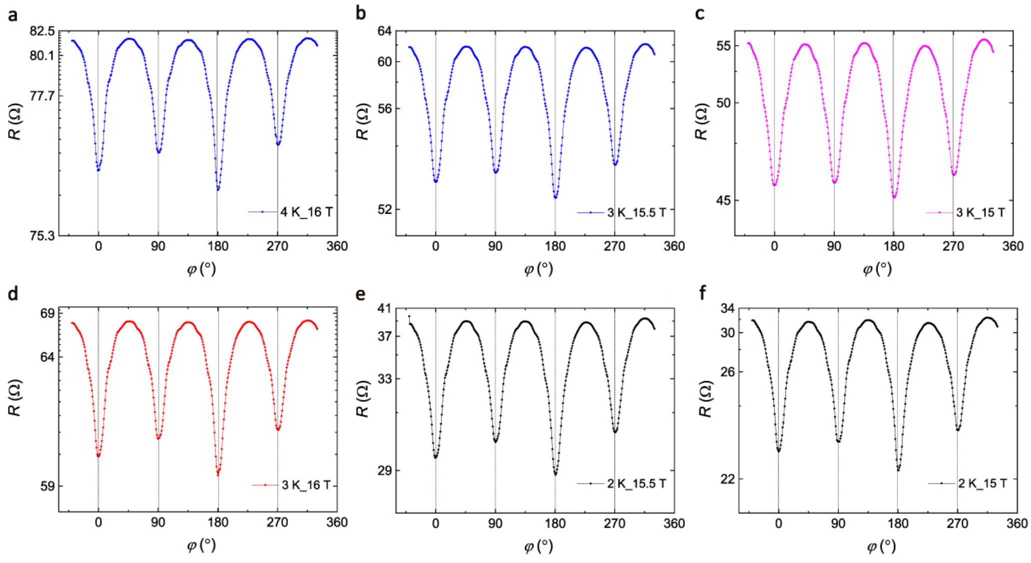

Fig. S13. Reproducible C_4+C_2 anisotropy at different temperatures and magnetic fields. Azimuthal angular dependence of magnetoresistance $R(\varphi)$ curves showing reproducible C_4+C_2 symmetry at 4 K and 16 T (**a**), 3 K and 16 T (**d**), 3 K and 15.5 T (**b**), 2 K and 15.5 T (**e**), 3 K and 15 T (**c**) and 2 K and 15 T (**f**). The logarithmic scale is

used on the resistance axis.

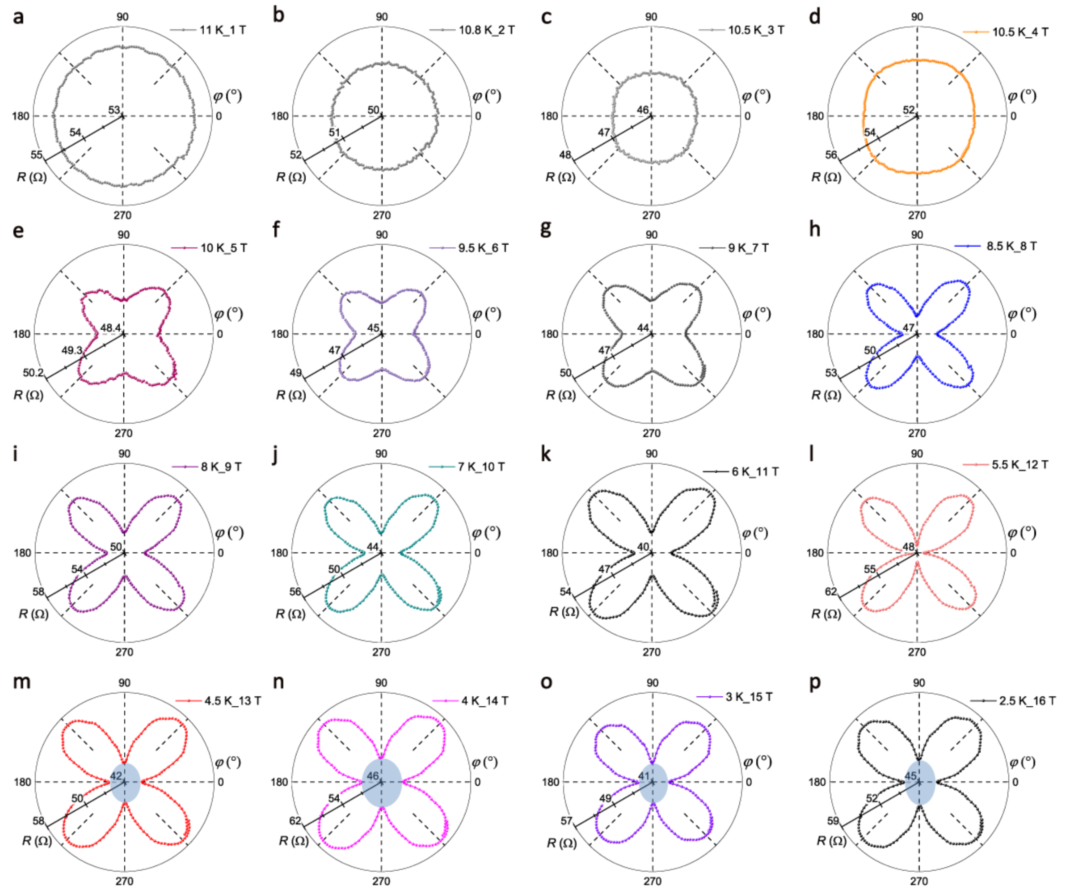

Fig. S14. *R*(*φ*) curves at different temperatures and magnetic fields of the superconducting state. *R*(*φ*) curves in the polar plots at different temperatures and magnetic fields from 1 T to 16 T (**a-p**), showing the evolution of the rotational symmetry from isotropic to C_4 symmetric and then to C_4+C_2 anisotropic. The temperatures and magnetic fields are labeled in the corresponding plots. The blue areas are guides to the eye, representing the C_2 anisotropy.

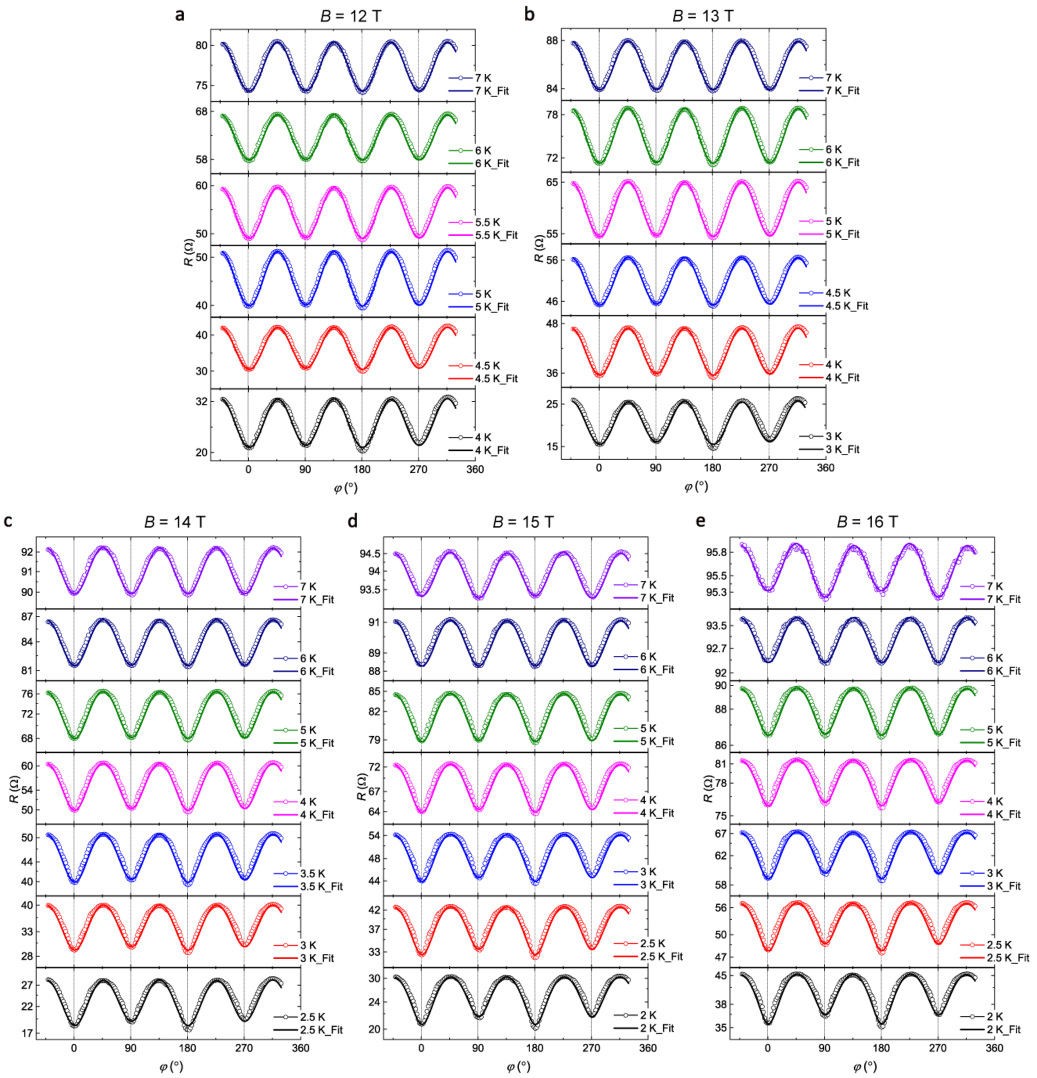

Fig. S15. Fit of the C_4+C_2 symmetric $R(\varphi)$ curves at different temperatures and magnetic fields. $R(\varphi)$ curves and the corresponding trigonometric function fits at different temperatures and magnetic fields from 12 T to 16 T (**a-e**). Each $R(\varphi)$ curve is fitted by the trigonometric function $R = R_{avg} + \Delta R_{C4}\times\sin(4\varphi) + \Delta R_{C2}\times\sin(2\varphi)$ to extract the C_2 component (ΔR_{C2}) and C_4 component (ΔR_{C4}). Here, the logarithmic scale is used on the resistance-axis in **c** to **e** to specifically demonstrate the C_2 symmetric feature.

III. Extra four-fold symmetric anisotropy of the normal state $R(\varphi)$ curves (referred to as C_4') observed in the large magnetic field regime (above 15 T)

In the superconducting state, the $R(\varphi)$ curves show C_4 or C_4+C_2 anisotropic features above approximately 4 T. The anisotropies disappear when the

superconductivity is destroyed by increasing temperature, indicating the anisotropies are related to the superconducting state. This relation can be confirmed by the absence of anisotropy in the normal state $R(\varphi)$ curves of the $Nd_{0.8}Sr_{0.2}NiO_2$ thin film, when the applied magnetic field is below 15 T (Fig. S16). Interestingly, when the applied field is above 15 T, the $R(\varphi)$ curves in the normal state show extra four-fold (C_4') anisotropy, which is much weaker, and has different orientations ($R(\varphi)$ maxima along *a*/*b*-axis) compared to the C_4 anisotropy in the superconducting state ($R(\varphi)$ minima along *a*/*b*-axis). Therefore, this extra C_4' anisotropy should have a different origin compared with the C_4 anisotropy (detailed comparison can be found in Fig. S17).

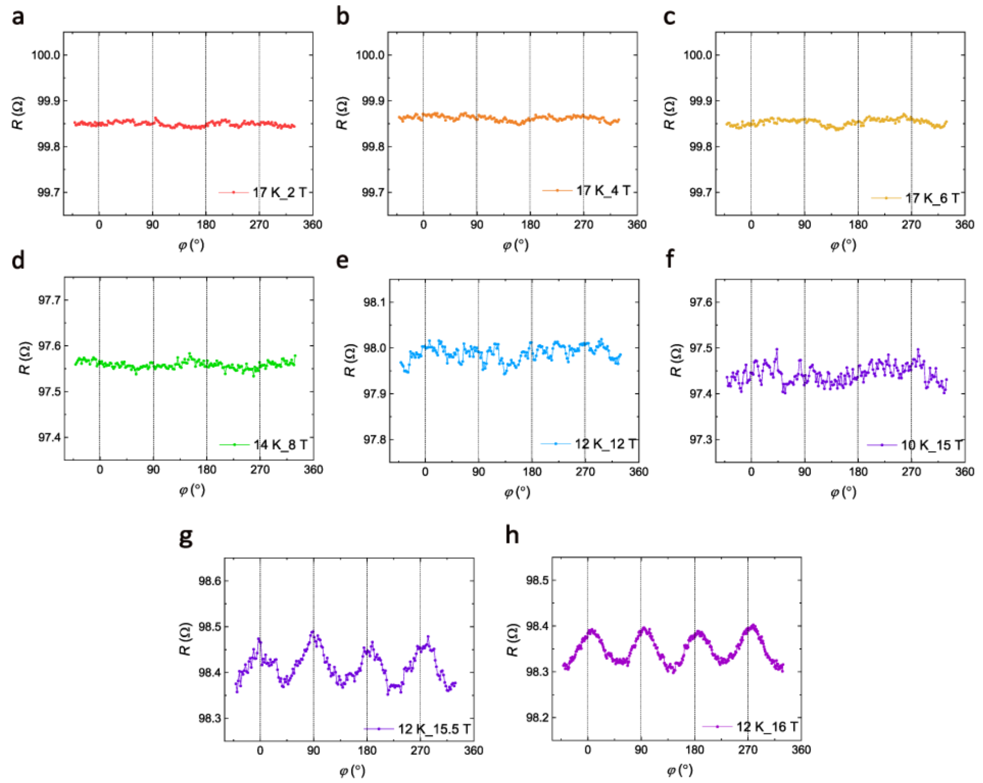

Fig. S16. C_4' anisotropy in the $R(\varphi)$ curves of the normal state. Rectangular plot of the $R(\varphi)$ curves measured under different magnetic field and temperature (**a-h**), where the $Nd_{0.8}Sr_{0.2}NiO_2$ thin film is in the normal state. The temperatures and magnetic fields are labeled in the corresponding plots. The $R(\varphi)$ curves are almost isotropic below 15 T, while the C_4' anisotropy emerges above 15 T.

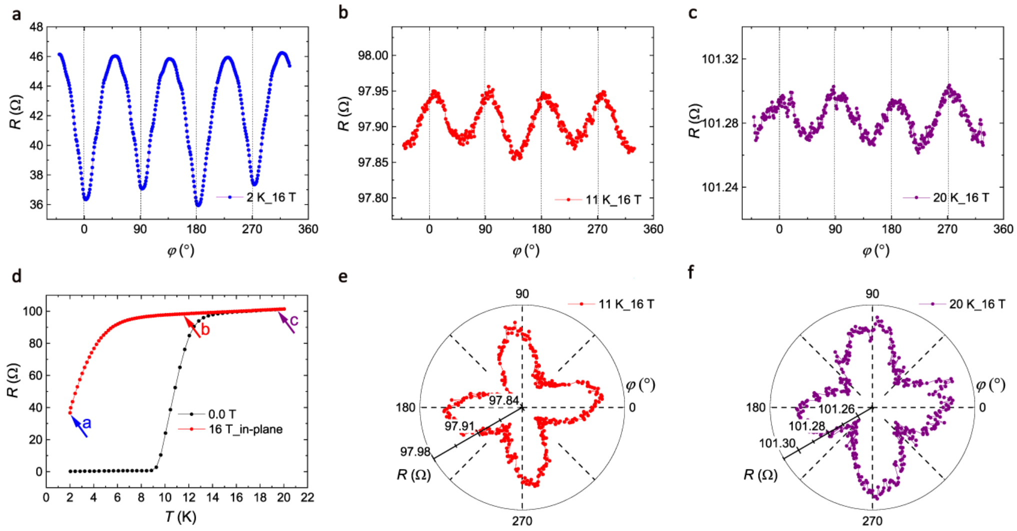

Fig. S17. C_4' anisotropy in the $R(\varphi)$ curves of the superconducting state and the normal state. a, Rectangular plot of the $R(\varphi)$ curve measured at 2 K and 16 T where the $Nd_{0.8}Sr_{0.2}NiO_2$ is in the superconducting transition, showing C_4+C_2 anisotropy. **b** and **e**, Rectangular plot (**b**) and polar plot (**e**) of the $R(\varphi)$ curves measured at 11 K and 16 T where the $Nd_{0.8}Sr_{0.2}NiO_2$ is in the normal state, showing C_4' anisotropy with different orientations and two orders of magnitude smaller compared with (**a**). **c** and **f**, Rectangular plot (**c**) and polar plot (**f**) of the $R(\varphi)$ curves measured at 20 K and 16 T where the $Nd_{0.8}Sr_{0.2}NiO_2$ is in the normal state, showing C_4' anisotropy. **d**, $R(T)$ curves under 0 T and 16 T. Three arrows indicate the corresponding temperature where (**a**), (**b**) and (**c**) are measured. The $R(\varphi)$ curves shown here are measured with a current of 5 μA. Compared with the C_4 anisotropy of the superconducting state, the C_4' anisotropy in the normal state is much weaker (two orders of magnitude weaker). The C_4' anisotropy show different orientations because it exhibits $R(\varphi)$ maxima at 0°, 90°, 180° and 270°, namely $R(\varphi)$ maxima along *a*/*b*-axis, while C_4 anisotropy exhibits minima along *a*/*b*-axis. Furthermore, the C_4' anisotropy is observed above 15 T, while the C_4 anisotropy emerges when the in-plane magnetic field is larger than 4 T. These results confirm that the C_4' anisotropy should have a different origin compared with the C_4 anisotropy. As demonstrated above, the C_4 anisotropy can be attributed to the unconventional superconductivity. In contrast, the C_4' symmetry with a smaller magnitude observed in

the normal state of the $Nd_{0.8}Sr_{0.2}NiO_2$ may be ascribed to the magnetic moment of the rare-earth Nd^{3+}. The C_4' symmetric $R(\varphi)$ curves in the normal state with a magnitude of 0.05% have ever been observed in the cuprates[1], similar to our observation of C_4' anisotropy in the normal state.

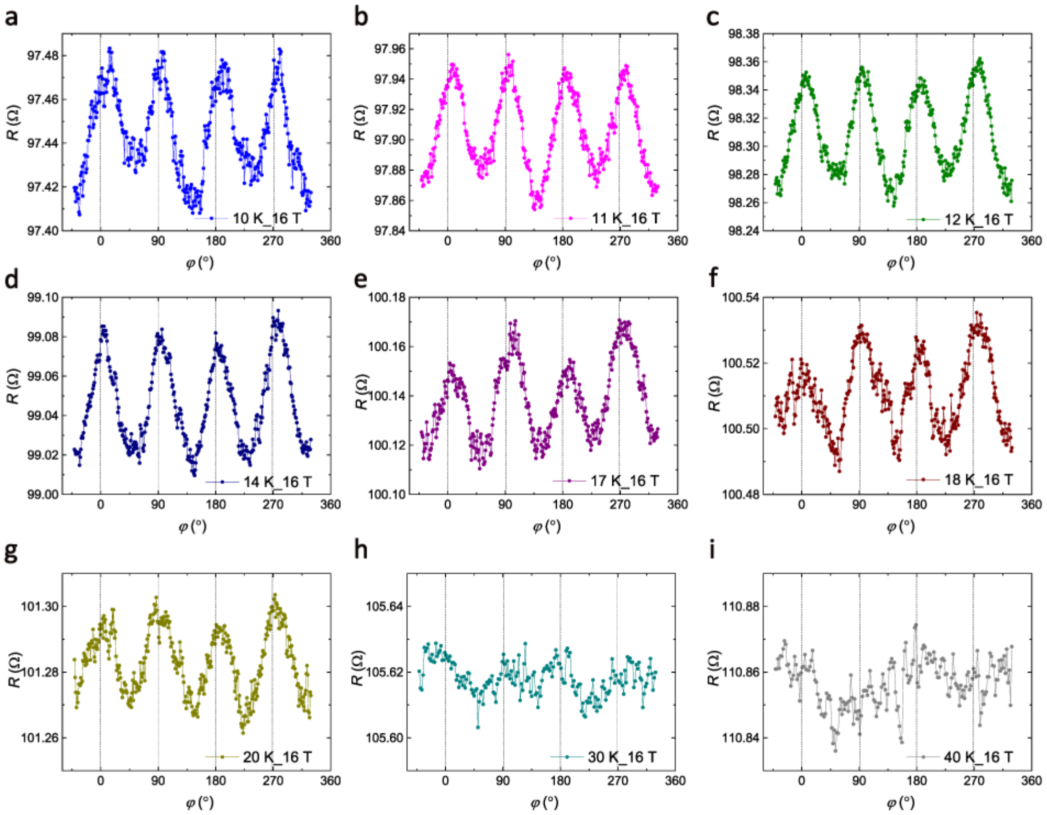

Fig. S18. C_4' anisotropy in the $R(\varphi)$ curves of the normal state. $R(\varphi)$ curves under 16 T at different temperatures in the rectangular plots (**a-i**), where the $Nd_{0.8}Sr_{0.2}NiO_2$ thin film is in the normal state. The temperatures and magnetic fields are labeled in the corresponding plots. The C_4' anisotropy smears out with increasing temperature, and is indistinguishable above 30 K. The excitation current is 5 μA.

Note that in our work the potential strain, inhomogeneity, underlying tetragonal crystal structure, and magnetic ordering of Nd^{3+} could not lead to the observed C_4 anisotropy, since the $R(\varphi)$ curves of the superconducting state below 4 T (Fig. S14 and Fig. S24**b**) and the $R(\varphi)$ curves of the normal state below 15 T (Fig. S16) are isotropic within the measurement resolution. To be specific, the potential strain, inhomogeneity

and the crystal structure should be independent of the external magnetic fields, and are not supposed to induce the magnetic field-dependent rotational symmetry breakings from isotropy (< 4 T) to C_4 (4 to 12 T) and then to C_4+C_2 (> 12 T) anisotropy in the superconducting states $Nd_{0.8}Sr_{0.2}NiO_2$ thin films. Besides, in all our samples, the $R(\varphi)$ minima (maxima) of the C_4 symmetry are fixed with the *a*/*b*-axis (45° to the *a*/*b*-axis), and all the C_2 anisotropies exhibit the same orientations along the *a*/*b*-axis of the $Nd_{0.8}Sr_{0.2}NiO_2$ thin films. The fixed orientations exclude the possibility that the anisotropies originate from the potential inhomogeneities, which should be different in different samples.

In particular, is it possible that the C_4 anisotropy in the superconducting state of the $Nd_{0.8}Sr_{0.2}NiO_2$ thin films can be attributed to the magnetic Nd^{3+} with $4f$ electrons located in the tetragonal crystal structure? Such a magnetic Nd-based hypothesis can be excluded as follows. First, we have observed that the C_4 anisotropy of the superconducting state disappears when the superconductivity is destroyed by increasing temperature, which unambiguously demonstrate that the C_4 anisotropy is associated with the unconventional superconductivity. Such a temperature-dependence cannot be explained by the magnetic Nd-based hypothesis, which is irrelevant to the superconducting transition. Second, the magnetic Nd-based hypothesis cannot explain the isotropic $R(\varphi)$ curves in the small magnetic field regime (< 4 T), and cannot explain the magnetic field-dependent rotational symmetry breaking from isotropy (< 4 T) to C_4 anisotropy (> 4 T). Third, the magnetic Nd-based hypothesis cannot explain our observation of intrinsic C_2 anisotropy, which breaks the C_4 rotational symmetry of the crystal structure. Fourth, this hypothesis cannot simultaneously explain the C_4' anisotropy of the normal state and the C_4 anisotropy of the superconducting state, because they have different manifestations and emerge in different magnetic field regimes. To be specific, the C_4' anisotropy is much weaker, and shows different orientation ($R(\varphi)$ maxima along *a*/*b*-axis) compared with the C_4 anisotropy ($R(\varphi)$ minima along *a*/*b*-axis). The C_4' anisotropy of the normal state is observed above 15 T, while the C_4 anisotropy of the superconducting state emerges when the in-plane

magnetic field is larger than 4 T.

IV. Reproducible results in sample S2

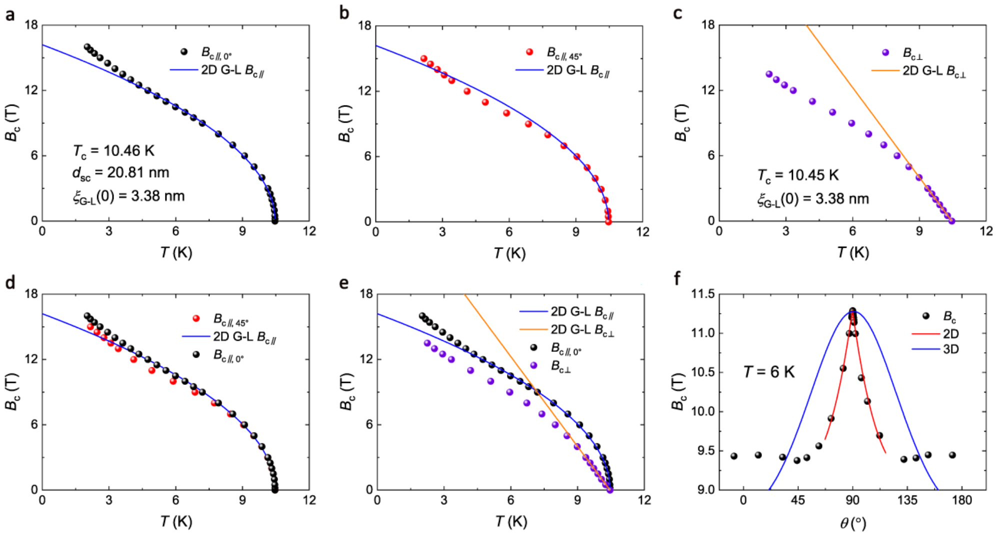

Fig. S19. Reproducible critical field behaviors in sample S2. Temperature dependence of the critical magnetic field measured along *a*/*b*-axis (**a**), the *ab* diagonal direction (**b**), and *c*-axis (**c**), **d**, Comparison between the $B_{c//, 0^o}^{50\%}(T)$ and $B_{c//, 45^o}^{50\%}(T)$. **e**, Comparison between $B_{c//, 0^o}^{50\%}(T)$ and $B_{c\perp}^{50\%}(T)$. The blue and the orange solid lines are the 2D G-L fittings of the $B_c(T)$ data near T_c. **f**, B_c at different θ angles at 6 K. The red solid line and blue solid line represent the theoretical fitting curves obtained by 2D Tinkham model and 3D anisotropic mass model, respectively.

It is noted that, in our two-dimensional Ginzburg-Landau (2D G-L) fit results, the coherence length ($\xi_{G-L}(0)$) is smaller than the superconducting thickness (d_{sc}). Currently, to our acknowledge, all the reported analyses of the infinite-layer nickelate superconductors based on the phenomenological 2D G-L formula yield consistent results that the $\xi_{G-L}(0)$ is smaller than the d_{sc}. The small $\xi_{G-L}(0)$ reminds us of the small coherence length in cuprate superconductors (basically 1 or 2 nm as previously summarized[2]). However, there is no established explanation so far regarding the small $\xi_{G-L}(0)$ in the nickelate superconductors. We hope our work will arouse more attention

and inspire further explorations in this topic. A brief summary of the $\xi_{G\text{-}L}(0)$ and d_{sc} of the infinite-layer nickelate superconductors is shown in Supplementary Table 1.

	Coherence length (nm)	Superconducting thickness (nm)	Sample
S1 in our work	3.45	19.53	$Nd_{0.8}Sr_{0.2}NiO_2$
S2 in our work	3.38	20.81	$Nd_{0.8}Sr_{0.2}NiO_2$
Nature **572**, 624 (2019)	3.25	Not mentioned, but the film thickness ~ 11 nm	$Nd_{0.8}Sr_{0.2}NiO_2$
Nat. Phys. **17**, 473 (2021)	4.26	22.5	$Nd_{0.775}Sr_{0.225}NiO_2$
Adv. Mater. **35**, 2303400 (2023)	2.75	6.49	$La_{0.8}Sr_{0.2}NiO_2$

Supplementary Table 1. Summary of the coherence length and the superconducting thickness of the infinite-layer nickelates obtained through 2D G-L formula.

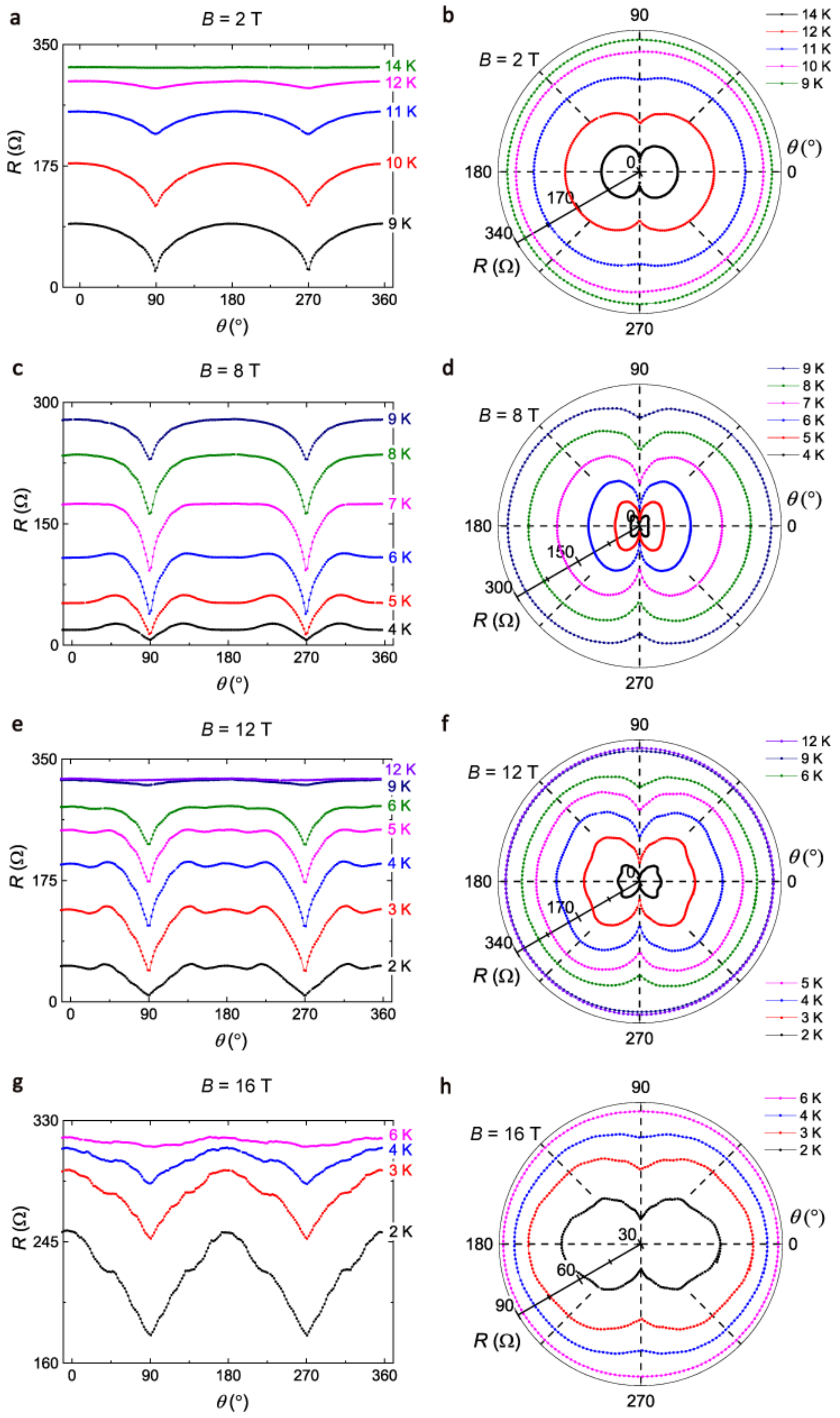

Fig. S20. Reproducible quasi-2D anisotropy in sample S2. Polar angular dependence of magnetoresistance $R(\theta)$ for sample S2 at different temperatures under 2 T (**a** and **b**), 8 T (**c** and **d**), 12 T (**e** and **f**), and 16 T (**g** and **h**). The left panels show the rectangular

plots and the right panels show the corresponding polar plots.

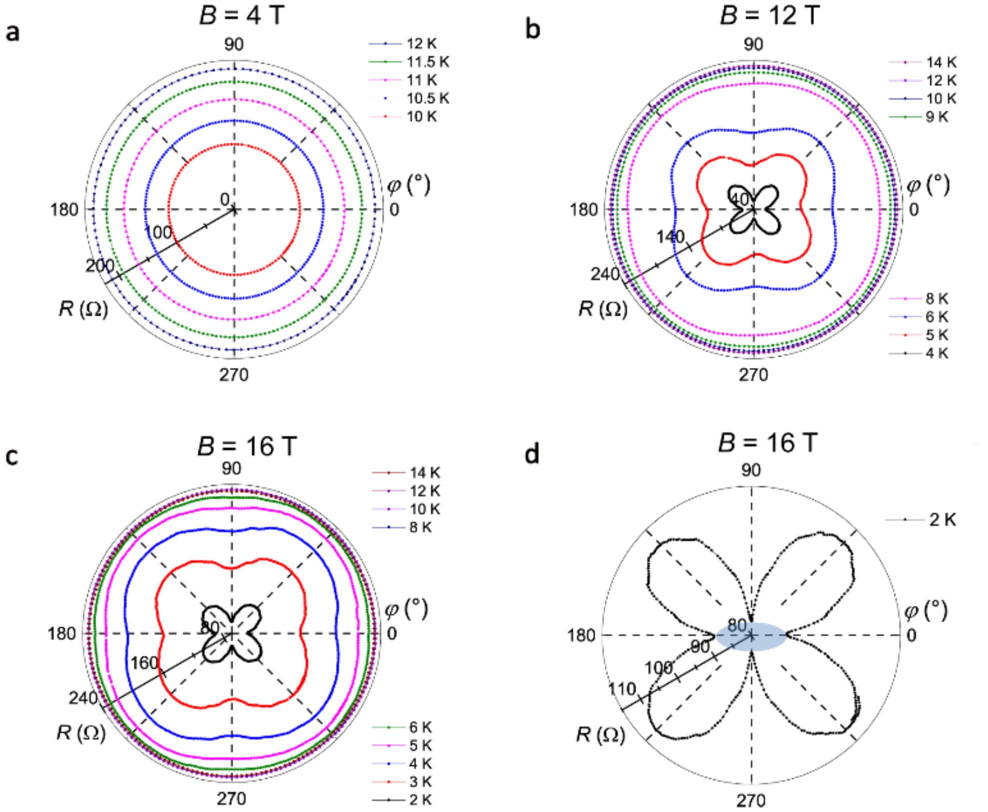

Fig. S21. Reproducible $R(\varphi)$ behaviors in sample S2. Azimuthal angular dependence of magnetoresistance $R(\varphi)$ for sample S2 at different temperatures under 4 T (**a**), 12 T (**b**), 16 T (**c**) in the polar plots, showing nearly isotropy, C_4 anisotropy, and C_4+C_2 anisotropy, respectively. The $R(\varphi)$ curve at 2 K and 16 T is further plotted in **d**, where the C_2 anisotropy can be better resolved. The blue area is a guide to the eye, representing the C_2 anisotropy.

V. Coincidence of the disappearance of C_4 anisotropy and the superconducting transition for a range of T_c

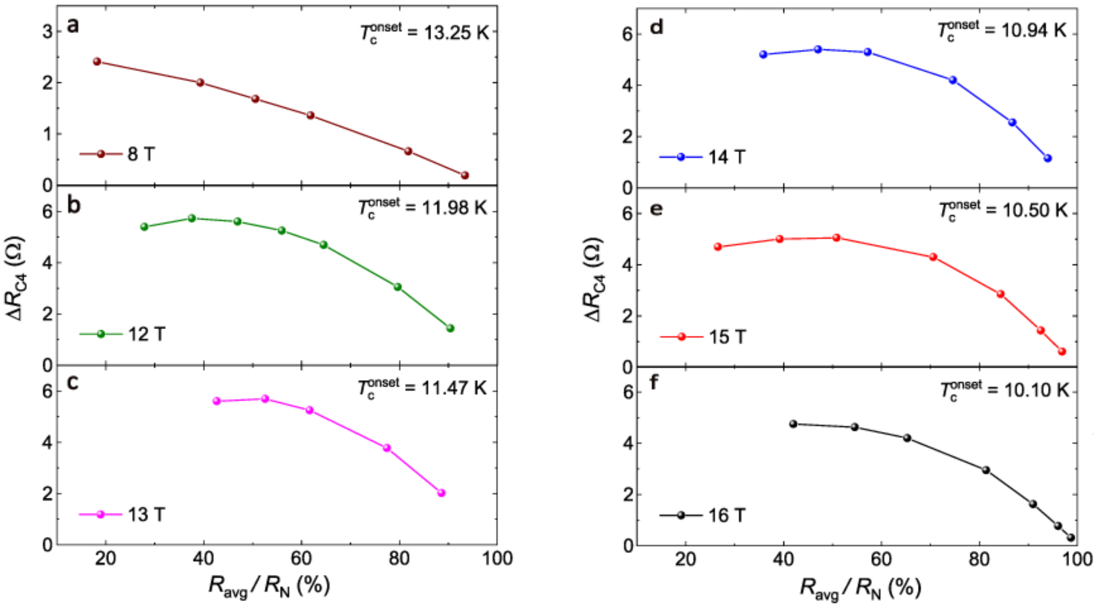

Fig. S22. Temperature-dependent C4 anisotropy components under different magnetic fields in sample S1. a to **f**, Four-fold components ΔR_{C4} versus the ratio between the averaged magnetoresistance and the normal state resistance (R_{avg}/R_N) under different in-plane magnetic fields. The T_c^{onset} varies with applied magnetic fields and is labelled in each panel. The ΔR_{C4} and R_{avg} values are extracted by trigonometric function fitting. When the R_{avg}/R_N value is getting close to 100%, the temperature is getting close to the onset superconducting transition temperature (T_c^{onset}), and the $Nd_{0.8}Sr_{0.2}NiO_2$ thin film is approaching the normal state and losing the superconductivity. The amplitudes of the C_4 anisotropy are gradually diminishing, showing a tendency that the C_4 anisotropy disappears just when the superconductivity is destroyed at higher temperatures, which strongly indicates that the C_4 anisotropy is associated with the superconducting state for T_c^{onset} ranging from 10.10 K to 13.25 K. This figure is modified from Fig. 2 in the main text.

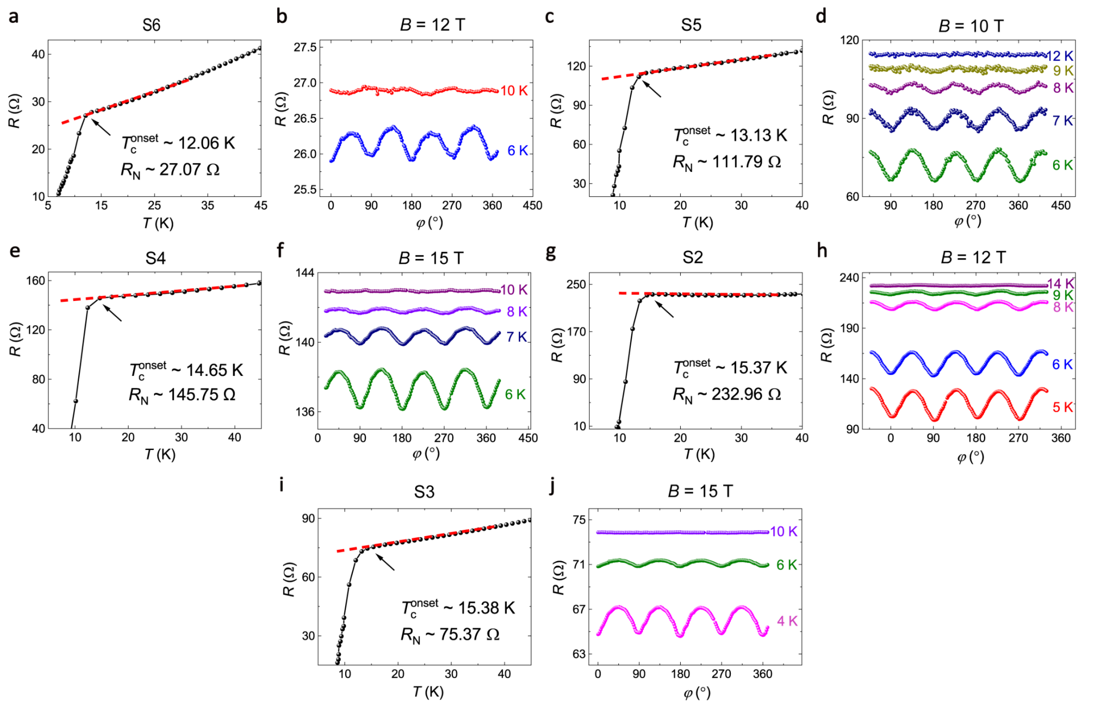

Fig. S23. Reproducible C4 anisotropy in more samples. Temperature-dependent resistance $R(T)$ (**a**, **c**, **e**, **g**, **i**) and $R(\varphi)$ (**b**, **d**, **f**, **h**, **j**) curves obtained from different samples S6 (**a** and **b**), S5 (**c** and **d**), S4 (**e** and **f**), S2 (**g** and **h**), S3 (**i** and **j**), whose T_c^{onset} (B = 0) varies from 12.06 K to 15.38 K as labelled in the panels. The C4 anisotropy in the $R(\varphi)$ curves of the superconducting state gradually fades away with increasing temperature, and disappears when approaching the normal state of the samples for all the $Nd_{0.8}Sr_{0.2}NiO_2$ thin films with different T_c^{onset} (B = 0) values. The reproducible results for a range of T_c^{onset} indicate that the C4 anisotropy is associated with the superconducting state.

VI. Angular dependent measurements using a two-axis stage instrument

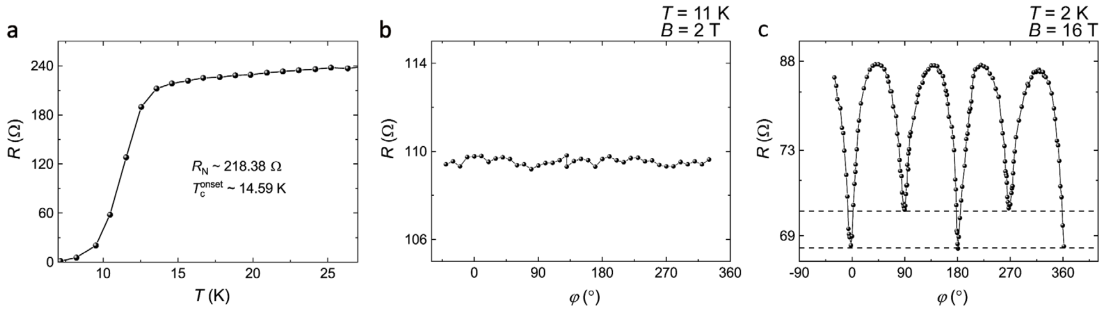

Fig. S24. Transport results using a two-axis rotator. a, Temperature-dependent resistance $R(T)$ of $Nd_{0.8}Sr_{0.2}NiO_2$ film sample S10 measured by the Corbino-disk configuration. **b**, **c**, Rectangular plots of azimuthal angle dependence of the magnetoresistance $R(\varphi)$ curves measured at 2 T and 11 K (**b**) and 16 T and 2 K (**c**), where the $Nd_{0.8}Sr_{0.2}NiO_2$ thin film is in the superconducting state. The $R(\varphi)$ curve under 2 T is isotropic within the experimental resolution. The $R(\varphi)$ curve under 16 T shows C_2 + C_4 rotational symmetry. A nearly perfect C_2 symmetric component can be determined by R (0°) ≈ R (180°) < R (90°) ≈ R (270°), as indicated by the black dashed lines.

VII. Reproducible C_2 symmetry superimposed on the C_4 symmetry in other samples.

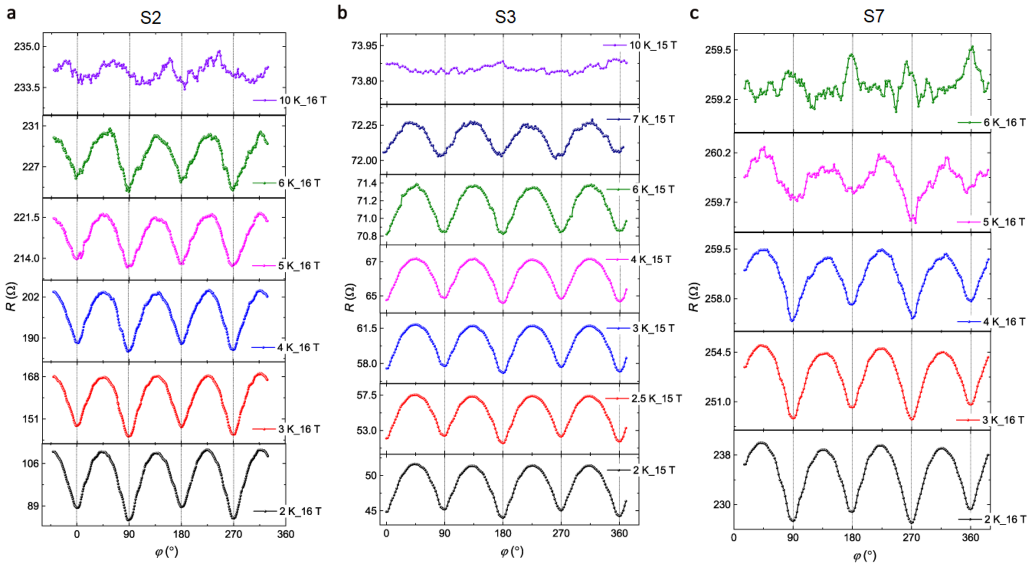

Fig. S25. Reproducible C_2 anisotropy superimposed on the C_4 symmetry in more samples. $R(\varphi)$ at different temperatures under 16 T from sample S2(**a**), S3(**b**), and S7(**c**), showing both the reproducible C_4 and C_2 symmetric features. The C_4 anisotropy is manifested as four minima at 0°, 90°, 180°, and 270° ($R(\varphi)$ maxima at 45°, 135°, 225° and 315°) (**a**, **b** and **c**). The C_2 anisotropy is manifested as R(0°) being larger than R(90°) (**a**) or R(0°) being smaller than R(90°) (**b** and **c**). The observations are consistent with the results of S1 shown in the main text.

In our measurements, there are slight differences in the resistance values between 0° and 180°, or 90° and 270°, implying a weak non-periodic signal. The similar non-periodicity showing an imperfect rotational symmetry was ever observed in previous angular dependent magnetoresistance reports on CsV_3Sb_5, $Ba_{0.5}K_{0.5}Fe_2As_2$, etc., even with the help of Corbino-disk configuration[3-5], which could be attributed to trivial mechanisms. For example, the misalignment of the magnetic field may give rise to the non-periodical and irreproducible features. Note that in our Corbino-disk measurements the non-periodic feature is supposed to have negligible impacts on determining the intrinsic C_4 and C_2 anisotropy of the $Nd_{0.8}Sr_{0.2}NiO_2$ thin films. First, a nearly perfect C_2 symmetric feature is confirmed through a two-axis stage rotator, which can basically eliminate the misalignment of the applied magnetic fields (Fig. S24**c**). Second, the signal amplitude of the non-periodic feature is quite weak, compared with the amplitude of the C_2 component, which can be as large as 5.0 Ω (Fig. S25**a**). Generally, in our measurements, the amplitude of the C_2 components is one order of magnitudes larger than the non-periodic feature. Third, the logarithmic scale is used on the resistance-axis to specifically demonstrate the C_2 symmetric feature in Fig. 2**e** and Fig. 3**c**. The small non-periodic feature is especially magnified by the logarithmic scale since it is close to the bottom of the axis. As shown in Fig. S25, in the linear scale plot, the non-periodic features are indistinguishable while the C_2 anisotropy is still evident. Fourth, in our experiments, the non-periodic feature shows evident irreproducibility among different samples, suggesting it is not an intrinsic property of our sample. The irreproducible characteristics are summarized in the Supplementary Table 2. On the contrary, the C_4 and C_2 anisotropic features exhibit strong reproducibility (Fig. S13 and S25), confirming the validity of our observations of the intrinsic C_4 and C_2 anisotropies.

Samples	Between 0° and 180°	Between 90° and 270°
S1	$R(0^o) > R(180^o)$	$R(90^o) < R(270^o)$
S2	$R(0^o) < R(180^o)$	$R(90^o) < R(270^o)$

S3	R(0º) > R(180º)	R(90º) > R(270º)
S7	R(0º) > R(180º)	R(90º) > R(270º)

Supplementary Table 2. The irreproducible characteristics of the non-periodic feature in different samples.

VIII. Exclusion of some other possibilities regarding the anomalous upturn of the critical field.

In our work, the emergence of a symmetry-breaking state in the low temperature and high magnetic field regime is inferred from the simultaneous occurrence of the spontaneous rotational symmetry breaking in $R(\varphi)$ curves (from C_4 to C_2) and the enhanced superconducting critical field behavior. Some other possible mechanisms of the anomalous upturn of the critical field can be excluded as follows.

The upturn of the $B_c(T)$ may be seen in the two-band superconductivity scenario, which, however, cannot explain our results. First, the temperature-dependence of critical current ($I_c(T)$) of our $Nd_{0.8}Sr_{0.2}NiO_2$ thin film is measured (Fig. S26**a** and Fig. S26**b**), which shows no kink features as expected for the two-band superconductivity [6,7]. Second, the two-band superconductivity fit fails to capture the $B_c(T)$ curves of our $Nd_{0.8}Sr_{0.2}NiO_2$ thin films, as shown in Fig. S26**c** and Fig. S26**d**. This is consistent with previous report[8], where the two-band superconductivity model in the dirty limit cannot reproduce the $B_c(T)$ behaviors in both in-plane and out-of-plane fields.

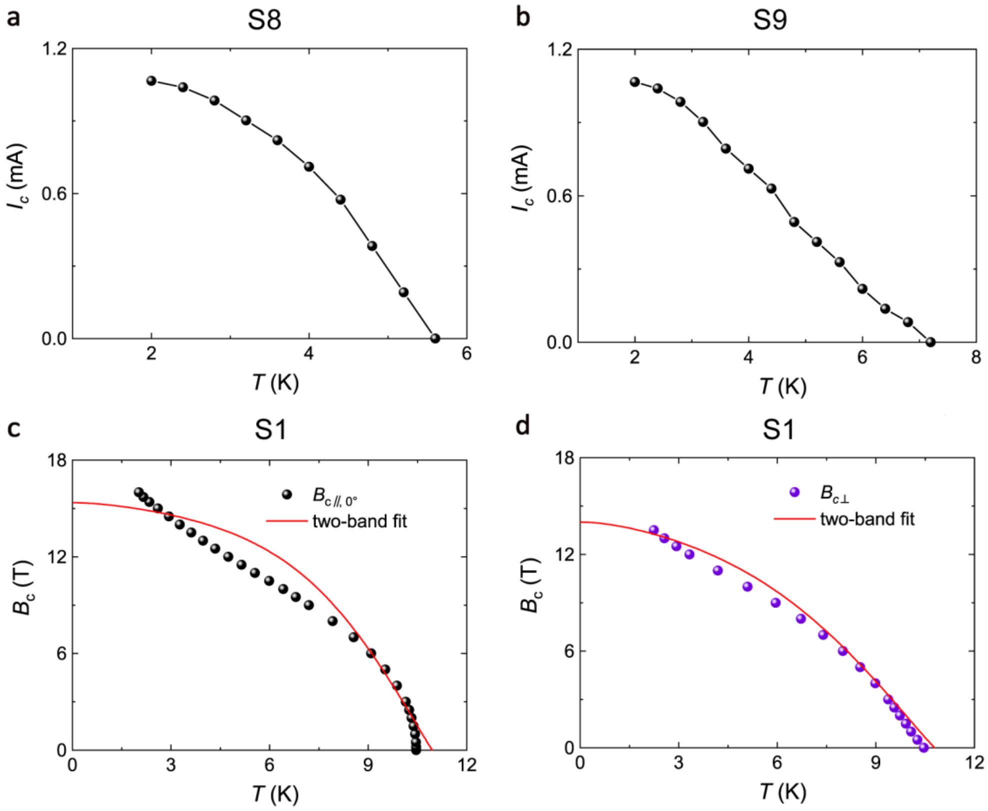

Fig. S26. $I_c(T)$ and $B_c(T)$ results. Temperature-dependent critical current $I_c(T)$ curves of sample S8 (**a**) and sample S9 (**b**). The $B_{c//,0°}(T)$ (**c**) and $B_{c\perp}(T)$ (**d**) and their corresponding two-band superconductivity fit curves of sample S1.

The Takahashi-Tachiki effect may exhibit an upturn in the $B_c(T)$ curves, which can be excluded as follows. First, the Takahashi-Tachiki effect applies to the superconductivity in artificially prepared superlattices with each thickness in the order of nanometers[9]. The required configuration differs from our $Nd_{0.8}Sr_{0.2}NiO_2$ single crystal thin films with a *c*-axis lattice constant around 3.4 Å. Second, the Takahashi-Tachiki effect predicts that the in-plane critical magnetic field $B_{c,//}(T)$ varies linearly in T near T_c[9]. However, the $B_{c,//}(T)$ follows the $(T_c\text{-}T)^{1/2}$-dependence near T_c in our experiments, which excludes the possibility of the Takahashi-Tachiki effect.

IX. The transport properties of $La_{0.8}Sr_{0.2}NiO_2$ thin films

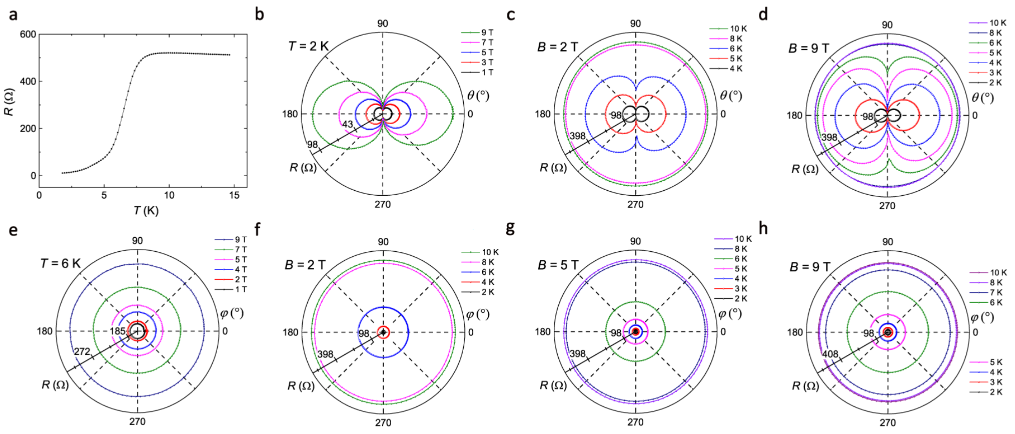

Fig. S27. The temperature-dependent resistance and the angular dependent magnetoresistance of La$_{0.8}$Sr$_{0.2}$NiO$_2$ film sample S1 measured by the Corbino-disk configuration. a, Temperature-dependent resistance $R(T)$ under zero magnetic field. **b**, **c**, **d**, Polar angular dependence of magnetoresistance $R(\theta)$ under different magnetic fields at 2 K (**b**) and at different temperatures under 2 T (**c**) and 9 T (**d**). Here, θ represents the angle between the magnetic field and the *c*-axis of the La$_{0.8}$Sr$_{0.2}$NiO$_2$. The $R(\theta)$ curves show two sharp dips in the low temperature, indicating the quasi-2D superconductivity. **e-h**, Azimuthal angular dependence of magnetoresistance $R(\varphi)$ curves under different magnetic fields at 6 K (**e**) and at different temperatures under 2 T (**f**), 5 T (**g**) and 9 T (**h**). Here, φ represents the angle between the magnetic field and the *a/b*-axis of the La$_{0.8}$Sr$_{0.2}$NiO$_2$. The $R(\varphi)$ curves are almost isotropic in different magnetic field and temperature regimes, differing from the behaviors of the Nd$_{0.8}$Sr$_{0.2}$NiO$_2$ thin films. Presumably, the Nd$_{0.8}$Sr$_{0.2}$NiO$_2$ is just around the border between the *s*-wave and (*d*+*is*)-wave superconducting symmetry, and thus the subtle balance between the competing pairing orders can be tilted by the magnetic fields. In contrast, the La$_{0.8}$Sr$_{0.2}$NiO$_2$ may locate in a different physical regime, preventing us from detecting the rotational symmetry breaking in the magnetic field range of 9 T. However, further investigations are still desired to confirm this scenario.

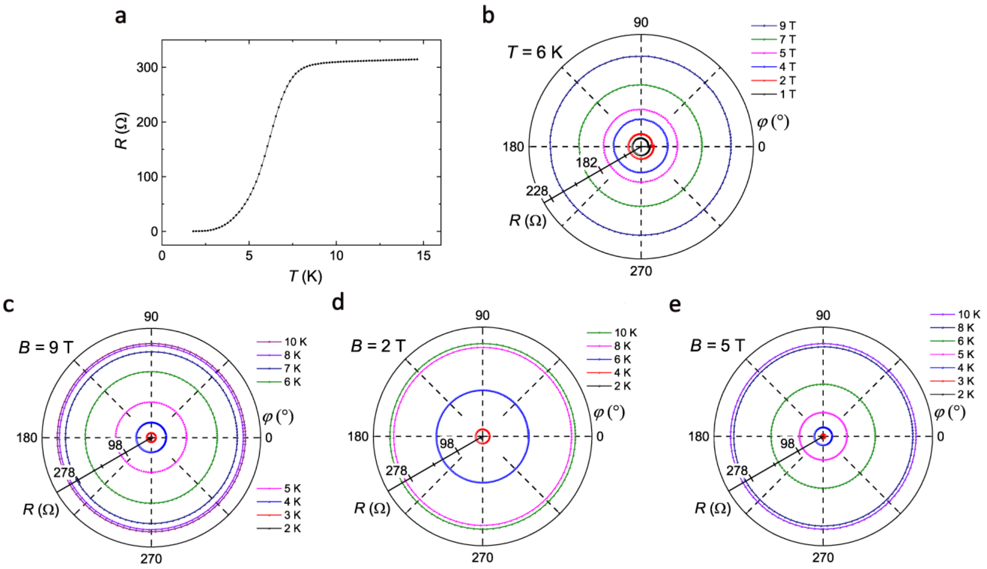

Fig. S28. The temperature-dependent resistance and the angular dependent magnetoresistance of $La_{0.8}Sr_{0.2}NiO_2$ film sample S2 measured by the Corbino-disk configuration. a, Temperature-dependent resistance $R(T)$ under zero magnetic field. **b-e**, $R(\varphi)$ under different magnetic fields at 6 K (**b**) and at different temperatures under 2 T (**c**), 5 T (**d**) and 9 T (**e**). The $R(\varphi)$ curves are almost isotropic in different magnetic field and temperature regimes.

Supplementary References

1. Jovanović, V. P., Fruchter, L., Li, Z. Z. and Raffy, H. Anisotropy of the in-plane angular magnetoresistance of electron-doped $Sr_{1-x}La_xCuO_2$ thin films. *Phys. Rev. B* **81**, 134520 (2010).
2. Shi, X., Dimitrov, I. K., Ozaki, T., Gu, G. and Li, Q. Quasi-two-dimensional fluctuations in the magnetization of $La_{1.9}Ca_{1.1}Cu_2O_{6+\delta}$ superconductors. *Phys. Rev. B* **96**, 184519 (2017).
3. Zhao, H. *et al.* Cascade of correlated electron states in the kagome superconductor CsV3Sb5. *Nature* **599**, 216-221 (2021).
4. Xiang, Y. *et al.* Twofold symmetry of c-axis resistivity in topological kagome superconductor CsV3Sb5 with in-plane rotating magnetic field. *Nat. Commun.* **12**, 6727 (2021).

5. Li, J. *et al.* Nematic superconducting state in iron pnictide superconductors. *Nat. Commun.* **8**, 1880 (2017).

6. Talantsev, E. F. *et al.* On the origin of critical temperature enhancement in atomically thin superconductors. *2D Materials* **4**, 025072 (2017).

7. Wan, P. *et al.* Orbital Fulde–Ferrell–Larkin–Ovchinnikov state in an Ising superconductor. *Nature* **619**, 46-51 (2023).

8. Wang, B. Y. *et al.* Isotropic Pauli-limited superconductivity in the infinite-layer nickelate $Nd_{0.775}Sr_{0.225}NiO_2$. *Nat. Phys.* **17**, 473-477 (2021).

9. Takahashi, S. and Tachiki, M. Theory of the upper critical field of superconducting superlattices. *Phys. Rev. B* **33**, 4620-4631 (1986).
